# Giant photoluminescence enhancement in tungsten-diselenide–gold plasmonic hybrid structures

**DOI:** 10.1038/ncomms11283

**Published:** 2016-05-06

**Authors:** Zhuo Wang, Zhaogang Dong, Yinghong Gu, Yung-Huang Chang, Lei Zhang, Lain-Jong Li, Weijie Zhao, Goki Eda, Wenjing Zhang, Gustavo Grinblat, Stefan A. Maier, Joel K. W. Yang, Cheng-Wei Qiu, Andrew T. S. Wee

**Affiliations:** 1 NUS Graduate School for Integrative Sciences & Engineering (NGS), National University of Singapore, 28 Medical Drive, Singapore 117456, Singapore; 2 Department of Physics, National University of Singapore, 2 Science Drive 3, Singapore 117542, Singapore; 3 Department of Physics, Imperial College London, London SW7 2AZ, UK; 4 Institute of Materials Research and Engineering, A*STAR (Agency for Science, Technology and Research), 3 Research Link, Singapore 117602, Singapore; 5 Department of Electrical and Computer Engineering, National University of Singapore, 4 Engineering Drive 3, Singapore 117583, Singapore; 6 Department of Electrophysics, National Chiao Tung University, Hsinchu 30010, Taiwan; 7 Physical Science and Engineering Division, King Abdullah University of Science and Technology, Thuwal 23955-6900, Kingdom of Saudi Arabia; 8 Department of Chemistry, National University of Singapore, 3 Science Drive 3, Singapore 117543, Singapore; 9 Centre for Advanced 2D Materials, National University of Singapore, 2 Science Drive 3, Singapore 117542, Singapore; 10 SZU-NUS Collaborative Innovation Center for Optoelectronic Science & Technology, Key Laboratory of Optoelectronic Devices and Systems of Ministry of Education and Guangdong Province, College of Optoelectronic Engineering, Shenzhen University, Shenzhen 518060, China; 11 Singapore University of Technology and Design, 8 Somapah Road, Singapore 487372, Singapore

## Abstract

Impressive properties arise from the atomically thin nature of transition metal dichalcogenide two-dimensional materials. However, being atomically thin limits their optical absorption or emission. Hence, enhancing their photoluminescence by plasmonic nanostructures is critical for integrating these materials in optoelectronic and photonic devices. Typical photoluminescence enhancement from transition metal dichalcogenides is 100-fold, with recent enhancement of 1,000-fold achieved by simultaneously enhancing absorption, emission and directionality of the system. By suspending WSe_2_ flakes onto sub-20-nm-wide trenches in gold substrate, we report a giant photoluminescence enhancement of ∼20,000-fold. It is attributed to an enhanced absorption of the pump laser due to the lateral gap plasmons confined in the trenches and the enhanced Purcell factor by the plasmonic nanostructure. This work demonstrates the feasibility of giant photoluminescence enhancement in WSe_2_ with judiciously designed plasmonic nanostructures and paves a way towards the implementation of plasmon-enhanced transition metal dichalcogenide photodetectors, sensors and emitters.

## Abstract (web summary)

Two-dimensional materials have excellent electrical properties, but poor luminescence limits their application in optoelectronics. Here, the authors demonstrate a plasmon-induced 20,000-fold enhancement in photoluminescence from tungsten diselenide suspended across a nanometre-scale gap.

## Graphical abstract

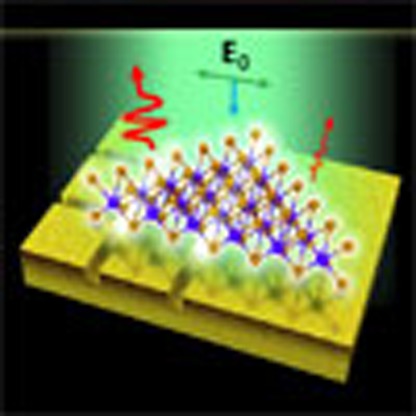

Crystalline monolayers of transition metal dichalcogenides (TMDCs) are direct bandgap two-dimensional (2D) material semiconductors that are promising as light-active materials for optoelectronic applications^1^,^2^. Recently, TMDCs have shown great potential in ultrafast and ultrasensitive photodetectors and as ultrathin light absorbers and emitters^3^,^4^,^5^. However, their application in photonic devices is limited by their low absolute photoluminescence (PL) caused by low quantum efficiency and weak absorption^6^. Fortunately, significant enhancement is afforded by integrating noble metal nanostructures supporting localized surface plasmons^7^,^8^,^9^,^10^,^11^,^12^,^13^ and propagating surface plasmon polaritons (SPPs)^14^,^15^.

Tungsten diselenide is a promising material in creating electrically excited light-emitting diodes^1^ and heterojunctions with MoS_2_ (ref. 5). It proves to be a promising emitter at ∼750 nm wavelength with a quantum yield that is 2 orders of magnitude larger than that of MoS_2_ (refs 16, 17). However, PL enhancements from WSe_2_-plasmonic hybrid nanostructure have yet to be investigated. Recently reported enhancements in MoS_2_ are potentially close to the ultimate limit that can be achieved from this material^9^,^18^. Although it is generally more challenging to achieve large enhancements from systems with a higher quantum yield, it is important to explore the ultimate limit in the PL enhancements that can be achieved in WSe_2_.

Here we report the surprisingly large PL enhancement factor (EF) of ∼20,000-fold from a single crystal monolayer of WSe_2_. WSe_2_-gold plasmonic hybrid nanostructures were created by suspending the monolayer over sub-20-nm-wide trenches that support lateral gap plasmons. By systematically tuning the gap plasmon resonances to the 633-nm pump laser, a close correlation was observed between the local near-field intensity, the Purcell factor and the measured PL enhancements. This paper sheds light on the utility of lateral gap plasmons in promoting excitation at the pump laser wavelength and emission at 750 nm, providing an effective way to obtain giant PL in TMDCs. Distinct from previous plasmon-enhanced structures^8^,^9^,^12^,^13^, our hybrid plasmon-enhanced design provides full access to the top surface of WSe_2_, for example, for layering of other 2D materials, electrical top contacts, chemical doping or optical waveguiding.

## Results

### WSe_2_-plasmonic hybrid nanostructures

**Figure 1:**
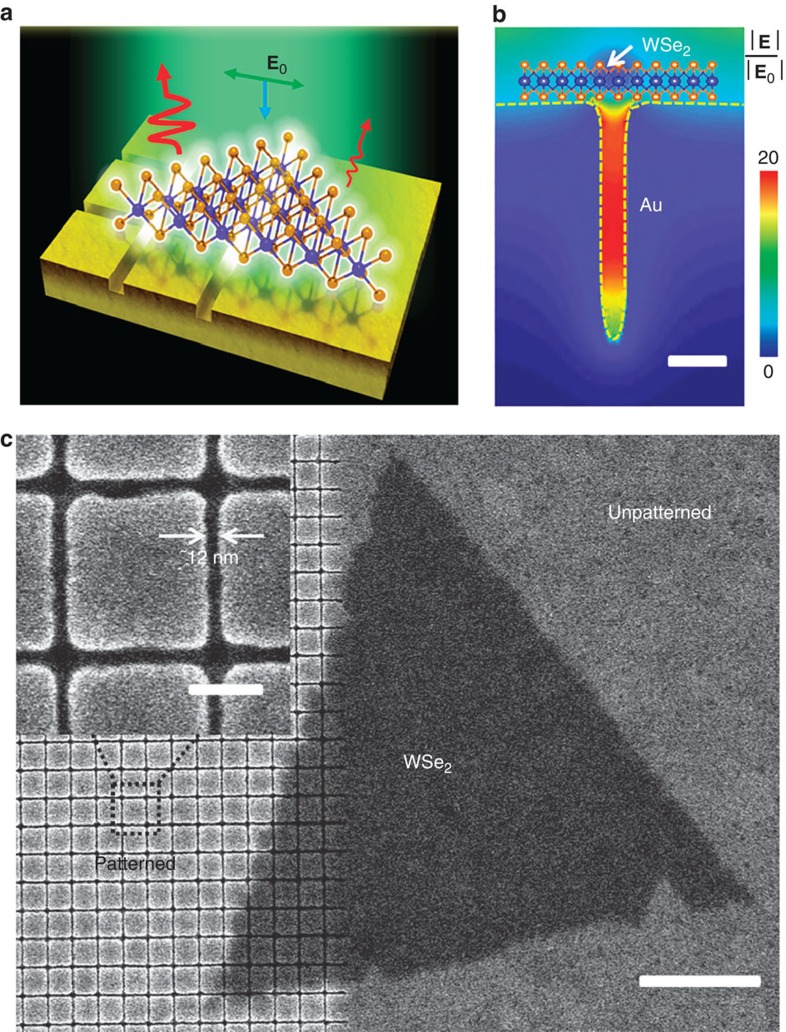
Schematic of WSe_2_-gold plasmonic hybrid structure with strong optical absorption. (**a**) Schematic of PL emission from a single crystal monolayer of WSe_2_ flake on a gold substrate. Part of the triangular flake rests on the patterned region of the substrate consisting of sub-20-nm-wide trenches. (**b**) Representative simulation of the electric field distribution of the lateral gap plasmons with a WSe_2_ monolayer flake suspended over a single trench. The polarization of the incident laser field is across the gap. The dashed yellow line denotes the boundary between air and gold. The scale bar, 20 nm. (**c**) Representative Scanning electron micrograph (SEM) image of WSe_2_ on square arrays with a trench width of 12 nm (Patterned) and unpatterned gold film (Unpatterned). The scale bars in the main figure and the inset, 1 μm and 100 nm, respectively.

A schematic of the investigated system is shown in Fig. 1a. We transferred chemical vapour deposition (CVD)-grown WSe_2_ monolayer flakes (Supplementary Note 1) onto gold substrates onto which trenches as narrow as sub-20 nm have been patterned. This sample enables a direct comparison of PL emission from the same single crystal WSe_2_ flake, where regions over unpatterned gold were taken as the base reference. The gold substrate was prepared by a recently reported template-stripping method based on nano-patterned silicon templates (Supplementary Fig. 1 and Supplementary Note 2)^19^. These structures have advantages over other commonly used gold nano-bowties, nano-rods or nano-discs made by electron-beam lithography (EBL)^8^,^12^,^13^, where template-stripping produces ultra-smooth surfaces, critical for reducing damping losses due to the reduction of plasmon scattering^20^,^21^. Moreover, we do template-stripping right before the transfer process of WSe_2_ so as to reduce contamination from adsorbed air-borne molecular species and preserve the hydrophilic nature of freshly stripped gold^22^.

The trenches in gold support strong field enhancements as shown in the finite-difference time-domain (FDTD) simulation results in Fig. 1b, where a monolayer WSe_2_ flake is placed over the trench. The resonances are in the form of lateral gap plasmons in the sub-20 nm trenches with **E**-fields predominantly parallel to the plane of WSe_2_ to promote strong light absorption^23^. This resonance can be tuned to be matched with the pump laser wavelength by varying the pitch of the structures, which are critical for plasmon coupling with light to achieve optimal field confinement^24^,^25^. Figure 1c presents a scanning electron micrograph (SEM) image of a triangular WSe_2_ single crystal monolayer as transferred onto the gold substrate with a trench width of ∼12 nm.

### Observation of PL enhancement in WSe_2_

**Figure 2:**
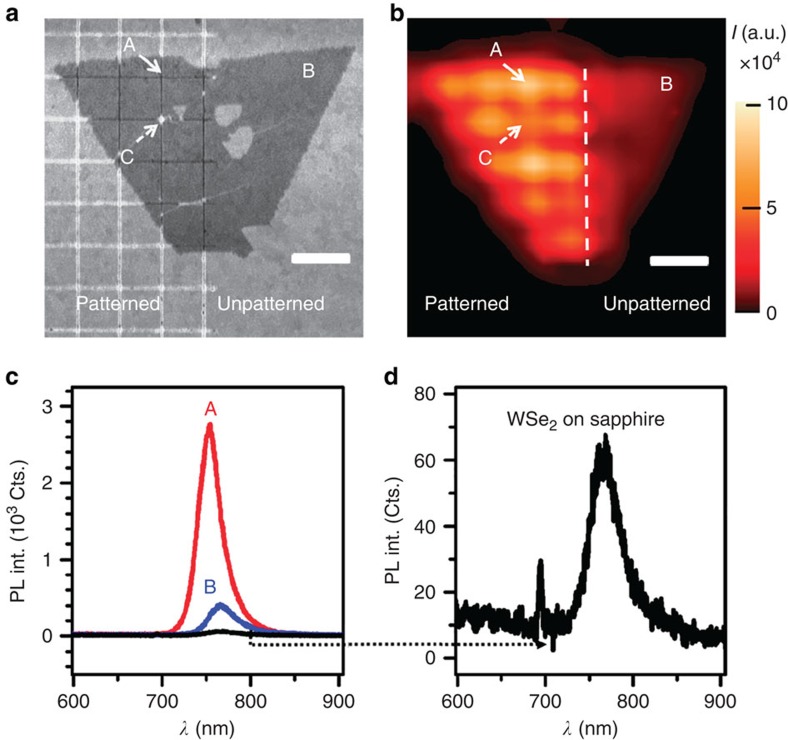
Characterization results of WSe_2_-gold plasmonic hybrid structure. (**a**) SEM image of a crystalline WSe_2_ monolayer flake transferred onto a template-stripped substrate with a pitch of 760 nm. ‘A' points to a portion of WSe_2_ suspended above an intersection of two underlying trenches, while ‘B' corresponds to the reference point, that is, WSe_2_ on unpatterned smooth gold. ‘C' points to a tear defect in the monolayer. The scale bar, 1 μm. (**b**) PL intensity (*I*) mapping on the WSe_2_-gold plasmonic hybrid structure showing larger signals from patterned regions and resolvable modulations in intensity. The intensity value at each pixel was obtained by integrating the PL spectrum across the spectrum window of 700–820 nm. A 532-nm pump laser was chosen here for a fine PL mapping resolution. (**c**) PL spectra from WSe_2_ on patterned gold nanostructures (A), unpatterned gold film (B) and the one from as-grown WSe_2_ on sapphire. Their PL peak energy (full-width-at-half-maximum) are 1.65 eV (63 meV), 1.63 eV (78 meV) and 1.61 eV (83 meV), respectively. (**d**) Zoom-in PL spectrum of WSe_2_ on sapphire. The laser power is 30 μw and integration time is 0.5 s.

Here we show that the lateral gap plasmons are able to enhance the PL emission significantly. A template-stripped gold substrate with a large pitch (760 nm) and 532-nm pump laser were chosen for this purpose so that the enhanced PL due to lateral gap plasmons in the trench could be spatially resolved. Figure 2a,b presents the SEM image and the corresponding PL mapping across the same WSe_2_ flake, so that it enables us to have a self-consistent comparison of PL enhancement from a single flake, thus avoiding potential variations in PL emission between different flakes. The PL experiment was carried out using a 532-nm pump laser with a power of 30 μW and the intensity value at each pixel of Fig. 2b was obtained by integrating the PL spectrum across the spectrum window of 700–820 nm. This PL mapping shows that the sub-20 nm trenches on gold substrate were able to enhance the PL emission considerably.

Figure 2c presents a quantitative comparison of PL spectra from WSe_2_ on patterned gold nanostructures (that is, Point A), unpatterned gold film (that is, Point B) and on sapphire. In this particular sample with a pitch of 760 nm, the 9-nm-wide trenches enhance the PL emission from WSe_2_ up to 37-fold as compared with the emission from WSe_2_ on sapphire. Moreover, PL emission from WSe_2_ on unpatterned gold film is enhanced by sevenfold as compared with the emission from WSe_2_ on sapphire (Fig. 2d), which might be due to the substrate-induced doping so as to reduce the non-radiative decay rates of the excitonic transitions^26^. In addition, the change in doping level could be induced from the downshift of Raman peak in WSe_2_ when transferred from sapphire onto gold substrate^27^ (Supplementary Fig. 2c). The weaker PL enhancement at Point C is due to the tear defect in the flake that is observable in the SEM image.

### PL enhancement mechanism

The physical processes involved in the PL enhancement are investigated next. The PL intensity of emitters is determined by its excitation rate (that is, absorption of the pump laser) and its emission efficiency (that is, Purcell factor). When the plasmon resonance of WSe_2_-gold nanostructures matches the wavelength of the pump laser, the excitation rate of WSe_2_ will be enhanced^28^, that is, *γ*_exc_∼|**E**_NF_|^2^ (near-field intensity enhancement). In addition, the plasmon resonance at the emission wavelength enhances the radiative decay rate and quantum efficiency via the Purcell effect^29^,^30^.

**Figure 3:**
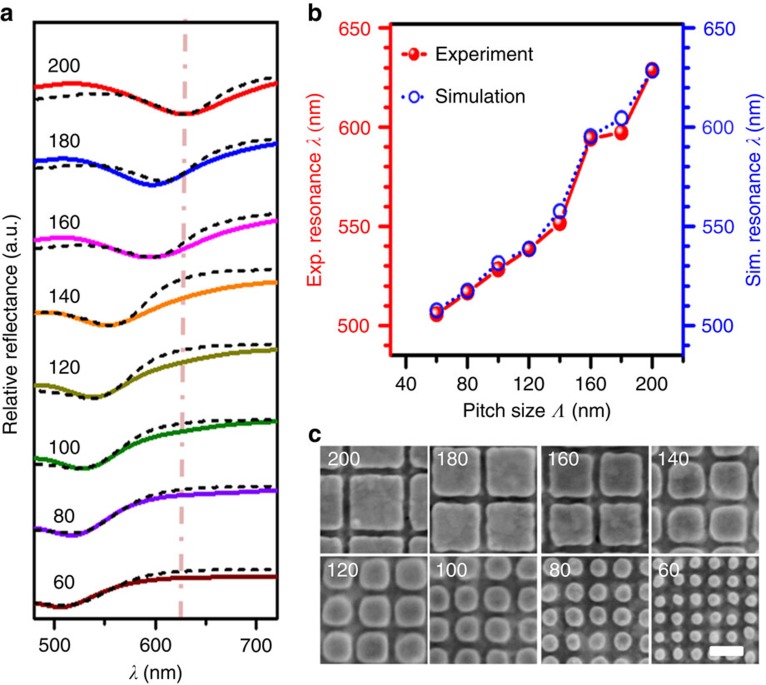
Optical characteristics of patterned gold nanostructures with different pitch sizes. (**a**) Relative reflectance spectra of patterned gold nanostructures with pitch sizes of 60–200 nm with respect to the unpatterned gold film. The solid and dashed curves present the experimental and simulated spectra, respectively. Legends denote the pitch size in units of nm for each pattern. The dash dot line indicates the position of the pump laser of 633 nm. (**b**) Experimental (Exp.) and simulated (Sim.) resonance wavelength of the patterned gold nanostructures as a function of pitch size. (**c**) SEM images of the template-stripped gold nanostructures with respective pitch sizes. The inset numbers denote the pitch sizes in the unit of nm. The scale bar in the SEM images, 100 nm.

The largest PL enhancement was achieved at the maximum of the product between the field EF at the excitation wavelength and the Purcell factor at the emission wavelength. Figure 3a presents the relative reflectance spectra as measured from gold nanostructures with different pitches, as normalized to the unpatterned gold film (see Methods). The plasmon resonance at the dips of the spectra red-shifted with increasing pitch. FDTD simulations were done for each nanostructure pitch, with nanostructure geometry adjusted to match the SEM images (Fig. 3c). The simulations show an excellent agreement with the measured spectra, as seen in Fig. 3a. Moreover, the dependence of the plasmon resonance on the pitch size is summarized in Fig. 3b, where the plasmon resonance red-shifts from 507 to 633 nm as the pitch size is increased from 60 to 200 nm. Resonance wavelengths beyond 633 nm were not obtained in our patterned nanostructures as the resonance wavelength for nanostructures with pitches >200 nm exhibited a blue shift due to a shallower trench depth as caused by imperfections in the fabrication process (see Supplementary Fig. 3 and Supplementary Note 3). Figure 3c presents the SEM images of the patterned trenches of different pitches. Well-defined square structures are observed in the patterned gold nanostructures for large pitch sizes of 140–200 nm, while the shape evolves into rounded disks as the pitch was reduced to 60 nm. This effect is due to the nanofabrication process, that is, proximity effects in EBL and micro-loading effects during etching that are more pronounced when the pitch is reduced.

**Figure 4:**
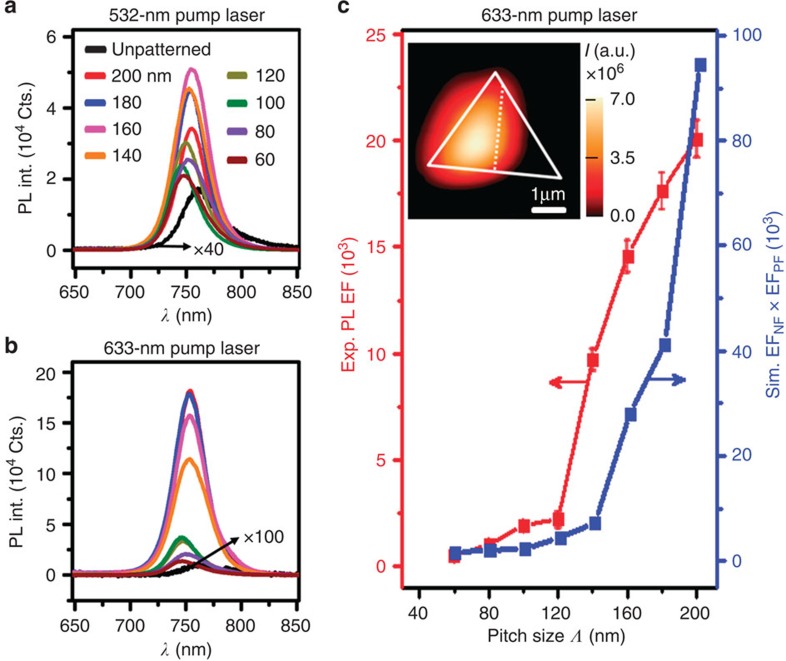
Photoluminescence of WSe_2_ on patterned gold nanostructures with different pitch sizes. (**a**) 532-nm pump laser. (**b**) 633-nm pump laser. (**c**) Experimental PL enhancement factor (EF) (left axis) excited by 633-nm laser (after correcting for the trench area fraction) and the simulated EF_NF_ (at 633 nm) × EF_PF_ (at 750 nm) (right axis), respectively. The data points and corresponding error bars are obtained, respectively, by calculating the mean value and s.d. of PL EF based on 10 experimental PL spectra for each nanostructure. The inset shows the PL mapping of a WSe_2_ monolayer flake sited on patterned gold nanostructure with a pitch of 200 nm (on the left of the dashed line) and unpatterned gold film (on the right of the dashed line). The intensity value at each pixel was obtained by integrating the PL spectrum across the spectrum window of 700–820 nm. The solid line represent the shape of the flake and the dashed line represents the boundary between the patterned and unpatterned substrate. The laser power was 30 μW and integration time was 0.5 s.

Both 532-nm and 633-nm pump lasers were used to investigate the effect of plasmon-enhanced PL emission from monolayer WSe_2_. Figure 4a,b presents the corresponding PL spectra as extracted from the PL mapping. We note that the 633-nm pump laser gives a higher PL emission as compared with the 532-nm pump laser. Despite the higher intrinsic absorption at 532 nm as compared with that at 633 nm in WSe_2_, excitation at 532 nm resulted in low field enhancements (Supplementary Fig. 4a,b) and low PL enhancements (Supplementary Fig. 4d), due to inter-band transitions of electrons in gold at 532 nm (ref. 31). Figure 4c presents the experimental PL EF (corrected by the trench area fraction) excited by the 633-nm laser and the product of the simulated EF of near-filed intensity and Purcell factor (see Methods) as a function of pitch size. Despite the higher density of trenches on the smaller pitch structures, on-resonance excitation with the pitch of 200 nm results in the strongest near-field intensity and Purcell factor enhancements, which were computed 1 nm above the gold substrate, that is, at the expected *z*-plane of WSe_2_. The s.d. in Fig. 4c was obtained by statistically analysing 10 spectra of WSe_2_ on each patterned gold nanostructure. Variations could be attributed to the slight trench size and shape variations of the patterned gold nanostructures, or optical non-uniformity among different triangular single crystals of WSe_2_.

**Figure 5:**
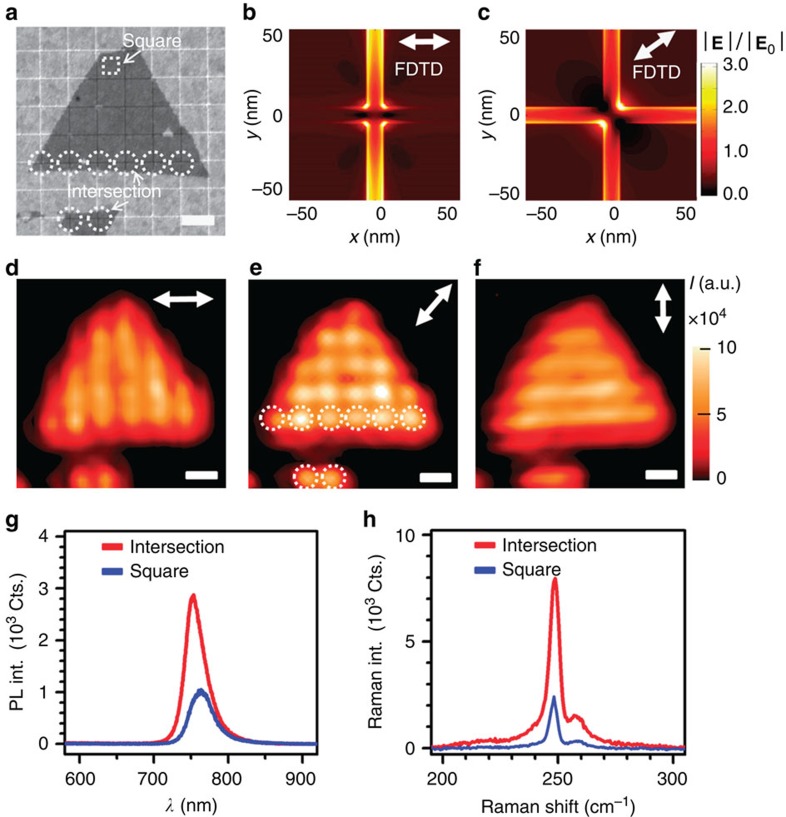
Polarization-dependent characteristics of PL emission from WSe_2_ flake on gold nanostructures with a pitch size of 920 nm and a pump laser of 532 nm. (**a**) SEM image of WSe_2_ flake on the gold nanostructures. The white dashed circles denote the region where the trenches in the gold film intersect and the dashed square denotes the centre of the square region. (**b**,**c**) Simulated electric field distribution at a plane 1 nm above the surface of patterned gold nanostructures with 0°- and 45°-polarizations, respectively. (**d**–**f**) Corresponding PL intensity mappings with polarization angles of 0°, 45° and 90°, respectively. The intensity value at each pixel was obtained by integrating the PL spectrum across the spectrum window of 700–820 nm. The white arrows show the polarization directions of the pump laser. (**g**,**h**) PL and Raman spectra of WSe_2_ taken at the intersection of gold trenches and square center, respectively. All the scale bars, 1 μm. The laser power and integration time for measuring PL (Raman) is 30 μW and 0.5 s, respectively (450 μW and 15 s).

As seen in Fig. 4c, the maximum PL enhancement was achieved at WSe_2_ on a nanostructure with the pitch of 200 nm. With the gap plasmon resonance tuned to the 633-nm pump laser wavelength, the integrated PL intensity from WSe_2_ on this gold nanostructure was enhanced up to 1,810-fold (without correction by the trench area fraction) compared with the reference on unpatterned gold film (see Supplementary Fig. 5a,b). Due to the small pitch size of 200 nm, the modulation of WSe_2_ PL intensity by the array of trenches could no longer be resolved in the PL mapping (see the inset of Fig. 4c). From Figs 1b and 5b as shown later on, we assume that the enhancements are still localized to WSe_2_ at the trenches, where this assumption will be supported by the PL mappings as shown in Fig. 5d,f later on. As measurements here were done with the pump laser polarized along the *x*-axis, fields are confined only within the trenches along the *y*-axis. Correcting for the small area occupied by these trenches, we obtain the maximum effective PL EF of ∼20,000 in WSe_2_ over the trench using the formula^9^:


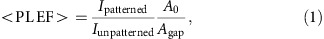


where *I*_patterned_ is the PL intensity from WSe_2_ on the patterned gold nanostructure and *I*_unpatterned_ is the PL intensity from WSe_2_ on the unpatterned gold film. *A*_0_ represents the excitation area of the laser spot size (*π* × 600^2^ nm^2^) and *A*_gap_ represents the area of the trenches (1.0 × 10^5^ nm^2^) perpendicular to the polarization direction of the laser within the laser spot (see detailed calculation in Supplementary Fig. 6 and Supplementary Note 4). To the best of our knowledge, this magnitude of enhancement has not been previously observed from 2D materials^7^,^8^,^9^,^10^,^11^,^12^,^13^,^14^,^15^.

Such a giant EF for the measured PL emission is due to the plasmon-enhanced excitation process and the plasmon-enhanced emission process. To be more specific, as shown in Supplementary Fig. 4b, the plasmonic nanostructure is able to enhance the excitation intensity by ∼310-fold, at the pump laser wavelength of 633 nm, when the pitch size is 200 nm. Furthermore, as shown in Supplementary Fig. 4c, the Purcell factor on this 200-nm-pitch nanostructure is able to be enhanced by ∼307-fold, as compared with the one on unpatterned gold film. Therefore, the experimental EF of PL emission could be predicted by the product of these two EFs, which is calculated to be more than 9 × 10^4^, as shown in Fig. 4c for the pitch size of 200 nm. In addition, we would like to mention that it is unlikely that phase transition of WSe_2_ is at play here, although there is direct contact between WSe_2_ and gold plasmonic structures. The semiconducting 2H to metallic 1T phase transition, which has only been observed in high-vacuum and low-temperature conditions^32^, has associated Raman and PL quenching effects^33^. However, we observed strong PL enhancement instead of quenching.

### Polarization dependence of PL emission

Next, we investigate the polarization-dependent characteristics of the PL emission from WSe_2_ on patterned gold nanostructures. To resolve the PL-enhanced regions, we chose a 920-nm-pitch substrate and a 532-nm pump laser to do the PL mapping. Here we choose the 532-nm pump laser to do the PL mapping simply because the 532-nm pump laser has a smaller spot size due to the shorter wavelength, as compared with the 633-nm pump laser. As a result, the imaging resolution of the PL mapping as obtained by the 532-nm pump laser is much clearer to show the enhanced PL emissions from trench regions. Figure 5a shows an SEM image of the sample. Figure 5b,c presents the simulated electric field distributions of lateral gap plasmons at the intersection of two trenches with 0°- and 45°-polarizations for the excitation. The simulated electric field distributions of this nanostructure agree with the experimental PL mappings in Fig. 5d–f. The maximum field enhancement occurs in trenches perpendicular to the polarization direction (Fig. 5b) and at the intersection of the trenches under 45°-polarization (Fig. 5c). A plot of PL intensity intersecting several trenches indicates a laser beam waist of ∼600 nm (see Supplementary Fig. 7). These results provide further evidence that the highest PL enhancements are from WSe_2_ directly above the trenches, where the optical fields are localized. It is noted that when the pitch size is larger than the pump laser wavelength, both lateral gap plasmons at the trenches and SPP on the flat squares are excited. However, the intensity of the SPP is only 1/6 of that of the lateral gap plasmon (see Fig. 5b,c). Thus, the contribution to the PL enhancement of WSe_2_ still dominantly stems from the lateral gap plasmons supported by the trenches. Figure 5g,h presents the PL and Raman spectra of WSe_2_ on the intersection and on the square centre, respectively. Both Raman and PL intensities of WSe_2_ at the intersection are found to be larger than the ones at the square centre. The un-shifted Raman peak of WSe_2_ over trenches and square centre suggests that no strain is induced by the trench and thus it also confirms that the observed PL enhancement was not induced by strain^34^.

## Discussion

Furthermore, the WSe_2_-gold plasmonic hybrid nanostructure allows us to conveniently evaluate a recent claim in the literature^35^, which states that suspended 2D materials are expected to have larger PL than the counterparts on a substrate, because the substrate depopulates the density of states in 2D materials through charger transfer. Nevertheless, as shown in Fig. 5d,f, the gaps parallel to the pump laser polarization do not exhibit any stronger PL over its surrounding, although WSe_2_ is suspended here. Therefore, it suggests that the suspension nature of WSe_2_ by the trenches contributes little to the PL enhancement.

It should be noted here that the square geometry chosen has the advantage of being independent of the incident polarization. In other words, if the incident polarization is either purely *x*-polarized or *y*-polarized, we will achieve the same PL EF. Therefore, for any other polarizations in-between, we always could decompose the incident optical field into the respective *x*-polarized component and *y*-polarized component. Since the PL emission intensity is linear with the excitation intensity, the PL EF remains the same.

Last, we would like to mention that there were no observable local heating effects in our experiments. In hybrid structure of MoS_2_-gold nanoantenna, an increased absorption of the laser light at the nanoantenna may cause local heating so as to red shift and broaden the MoS_2_ PL emission peak. Simultaneously, the local heating might cause a reduction in PL intensity of MoS_2_ (ref. 12). In our experiment, the PL peak position and its full-width-at-half-maximum (FWHM) of WSe_2_ on both patterned and unpatterned gold film have no power dependence (Supplementary Fig. 8a–d). Hence, for the range of laser powers used, the integrated PL and peak intensity increase linearly with respect to the laser power (Supplementary Fig. 8e,f), showing no signs of local heating effect.

In summary, concentrating and manipulating the electromagnetic field in sub-20 nm trenches can achieve unprecedented PL enhancement of WSe_2_. Tuning the resonance of lateral gap plasmons to match with the pump laser wavelength effectively boosts the light–matter interactions in WSe_2_, thus enhancing the light emission efficiency of WSe_2_. A giant PL EF of ∼20,000 was observed in WSe_2_ when using the 633-nm pump laser. We expect further PL enhancements to be achieved through the design of double-resonance structures with directionality control. This work demonstrates an important method to enhance the PL of TMDCs since it could enable high-efficiency and high-quality photodetectors and sensors, where photon absorption and emission dictate the device performance. The incorporation of gold arrays with sub-20-nm-wide trenches onto WSe_2_ monolayers opens up a new platform for investigating novel electrical/optical properties in 2D materials, such as electroluminescence and second harmonic generation, speeding up the applications of novel optoelectronic devices.

## Methods

### Material growth and quality of WSe_2_

The triangular-shape monolayer WSe_2_ was grown on sapphire by the CVD method (Supplementary Note 1). This technique yields high-quality monolayer WSe_2_ with a crystal size of ∼4.5 μm (Supplementary Fig. 2a). The typical PL spectrum of a pristine monolayer WSe_2_ (Supplementary Fig. 2b) shows one pronounced emission peak at 768 nm (1.61 eV), that is, the A direct excitonic transition^36^,^37^,^38^. The Raman spectrum excited by 532-nm laser is shown in the inset of Supplementary Fig. 2b, where the two characteristic peaks for monolayer WSe_2_ on sapphire are at 248 cm^−1^ and 259 cm^−1^, which are from the degenerate 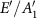 mode and 2LA(M) mode, respectively^39^.

### Fabrication of gold substrate and transferring of WSe_2_

The gold substrate consisting of patterned gold structure and unpatterned gold film was prepared by a template-stripping method (Supplementary Note 2). WSe_2_ flake was then transferred onto the gold substrate by a wet transfer approach (Supplementary Note 1). In comparison with the direct patterning of metallic nanostructures onto TMDCs by EBL, transferring WSe_2_ on top of plasmonic nanostructure has several advantages. First, it allows for direct incident laser irradiation on WSe_2_, instead of attenuated transmitted light irradiation on WSe_2_ through the plasmonic structures^13^. Second, it avoids the potential undesired damage or doping of WSe_2_ due to electron bombardment during electron-beam lithography process^40^. Last, the final structure will also be suitable for the direct access to the WSe_2_ flake, for example, for subsequent patterning or for contact to external leads.

### Characterizations

Atomic force microscope (AFM) images were measured by a BRUKER Dimension FastScan equipment. The optical reflectance spectra of the sample were then measured by using a CRAIC UV-VIS-NIR micro-spectrophotometer model QDI 2010 (equipped with a × 36 objective lens with numerical aperture (NA)=0.5). Moreover, SEM images were measured by FEI Verios 460. A confocal micro-PL set-up was used to excite the sample by using a 532-nm or 633-nm CW pump laser focused by a × 100 microscope objective lens (NA=0.65). PL emission was then collected by the same objective and detected by a nitrogen-cooled charge coupled device. The spot sizes of the 532-nm and 633-nm pump lasers were ∼600 nm and 1.2 μm, respectively. The PL mappings in Figs 2 and 5 were measured by the 532-nm laser as the smaller laser spot size of this gave a better PL mapping resolution. The background PL signals of pure gold nanostructures (Supplementary Figs 2e and 5c) were subtracted from all the PL spectra of WSe_2_ on patterned gold nanostructures. The PL EF is defined by equation (1).

### Numerical simulations

A commercial software package, Lumerical FDTD Solutions, was used to simulate the optical field distributions and Purcell factors. Periodic boundary conditions were used along *x*- and *y*-axes, while perfect-matched layer was used along *z*-axis. The finest mesh size was set to be 0.5 nm in the structure. The geometries of the metal nanostructures in the simulations were designed to match with the SEM images (Fig. 3c), with a trench depth of 75 nm. This fine adjustment in the geometry was done for every pitch, which explains the discontinuous jumps in the numerical data points (Fig. 3b and Supplementary Fig. 4). The simulated EF of near-filed intensity is defined as the ratio of the simulated |**E**|^2^ on top of the trench to the simulated |**E**|^2^ on the unpatterned gold film at pump laser wavelength. To estimate the emission enhancement, we first calculated the Purcell factors for dipoles located on top of the trenches of gold nanostructures and for dipoles on top of the unpatterned gold film^41^. The emission enhancement was then determined by the ratio of these Purcell factors, which can be obtained directly from the FDTD analysis. Note that the classical calculations of Purcell factors do take into consideration both radiative and non-radiative rates for the dipole in the different environments^42^. Classically, the non-radiative decay rates are due to Ohmic losses in the metallic structures adjacent to the dipole.

## Additional information

**How to cite this article:** Wang, Z. *et al*. Giant Photoluminescence Enhancement in WSe_2_-Gold Plasmonic Hybrid Structures. *Nat. Commun.* 7:11283 doi: 10.1038/ncomms11283 (2016).

## Supplementary Material

Supplementary Information Supplementary Figures 1-8, Supplementary Notes 1-4 and Supplementary References.
