# Supplementary material for: Giant photoluminescence enhancement in tungsten-diselenide–gold plasmonic hybrid structures
Source: Nat Commun. 2016 May 6;7:11283. doi: 10.1038/ncomms11283 (PMC4859068; doi:10.1038/ncomms11283)
Supplement: Supplementary Information — Supplementary Figures 1-8, Supplementary Notes 1-4 and Supplementary References. [file ncomms11283-s1.pdf]

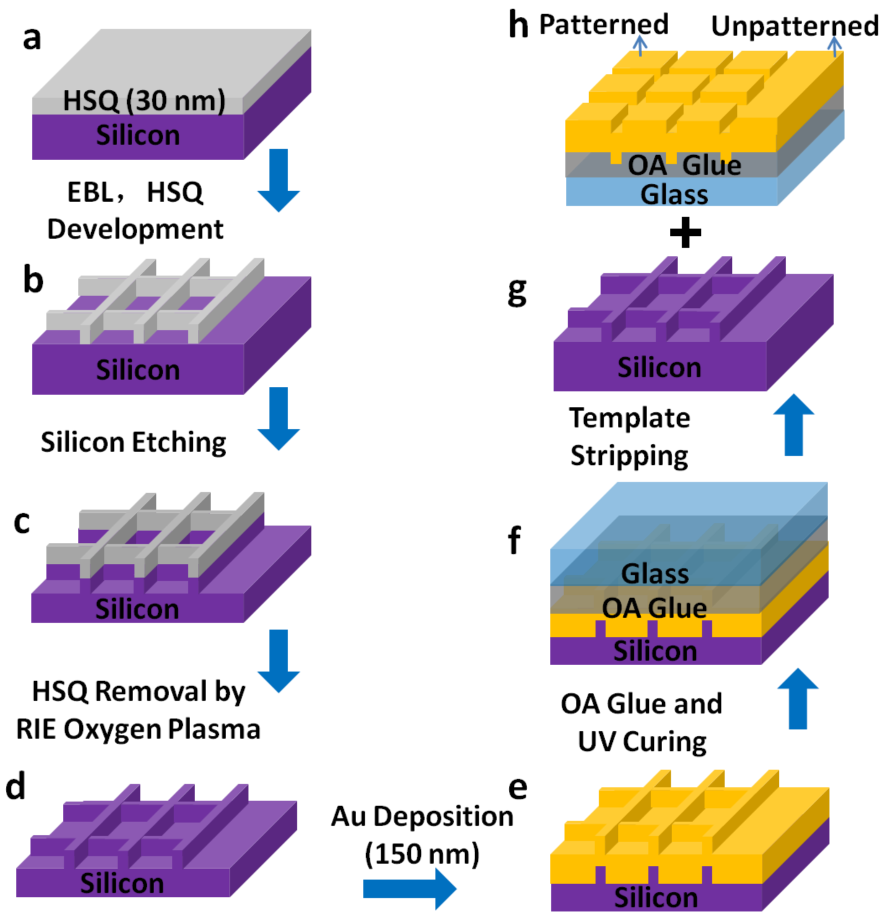

Supplementary Figure 1 | Detailed illustration on the fabrication process of template-stripped gold substrate. (**a**) Spin coating of hydrogen silsesquioxane (HSQ) resist onto the silicon substrate with a thickness of ~30 nm**.** (**b**) Electron beam exposure of HSQ resist with the design pattern and NaOH/NaCl salty development of HSQ resist. (**c**) Silicon etching by inductively coupled plasma (ICP). (**d**) Residue HSQ resist mask sputtered away by reactive-ion etching (RIE) oxygen plasma. (**e**) Deposition of 150-nm-thick gold film by using e-beam evaporator. (**f**) Drop casting of optical adhesive (OA) glue onto the gold film and followed by a glass coverslip. OA glue is then cured by UV irradiation for 30 minutes. (**g**)-(**h**) Template stripping process to fabricate the patterned gold nanostructures.

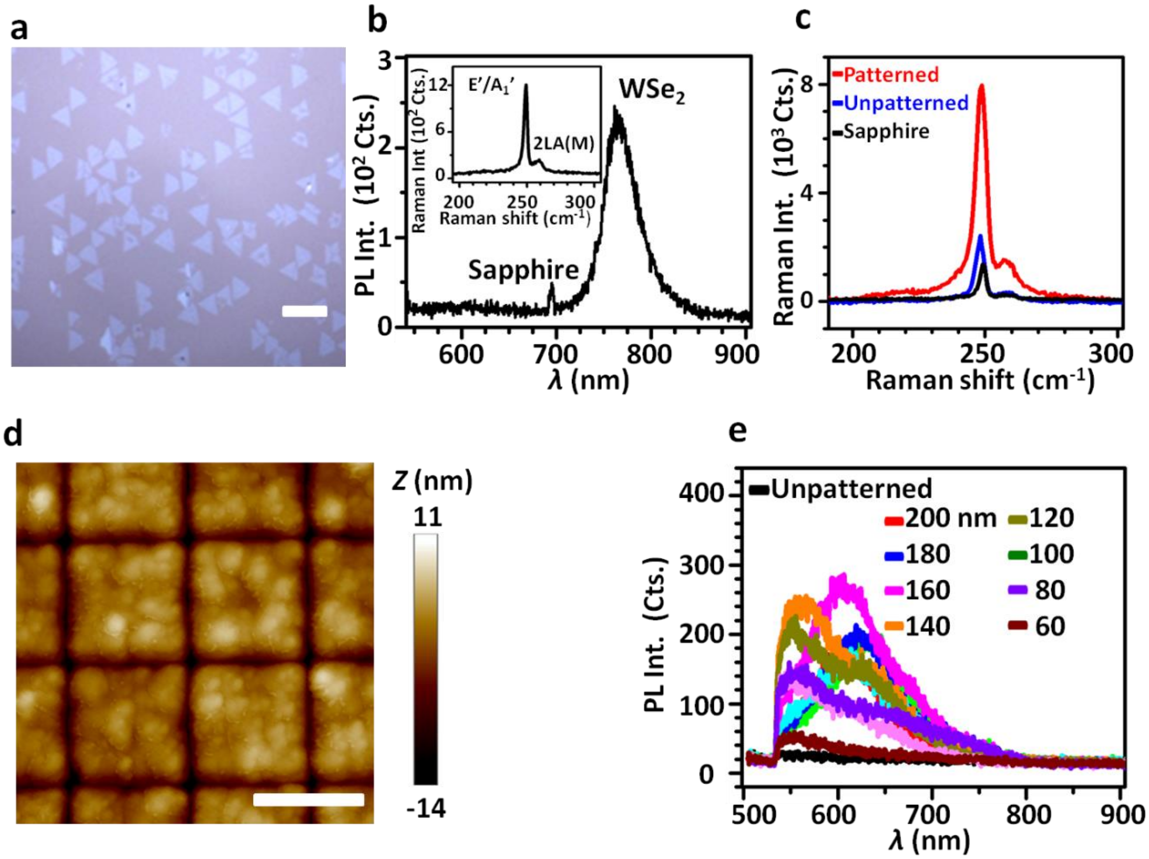

Supplementary Figure 2 | Characterization results of WSe_2 and gold substrate. (**a**) Optical microscope image of WSe_2 grown on sapphire. The scale bar is 10 µm. The black dots are nucleation points of WSe_2 in the growth process. (**b**) Raman and PL spectra of WSe_2 on sapphire as measured by 532-nm pump laser. The laser powers for measuring PL and Raman spectra were 110 µW and 450 µW respectively, and the corresponding integration times were 0.5 s and 15 s, respectively. (**c**) Comparison of Raman spectra from WSe_2 on patterned gold nanostructure, unpatterned gold film, and sapphire. The pump laser was 532 nm with the power of 450 µW. The integration time was 15 s. (**d**) AFM image of a patterned gold nanostructure after transferring WSe_2 monolayer flakes. The scale bar is 200 nm. The averaged root-mean-square (RMS) roughness value of the gold substrate prepared by template-stripping method can routinely achieve ~1.2±0.1 nm as characterized by atomic force microscopy (AFM). We would like to

mention that the fresh prepared template-stripped gold nanostructures, before transferring WSe_2 monolayers, have typical RMS roughness of 0.6 nm (Reference 2). (**e**) PL spectra of unpatterned gold film and bare patterned gold nanostructures excited by 532-nm laser. The power was 30 μW and the integration time was 0.5 s.

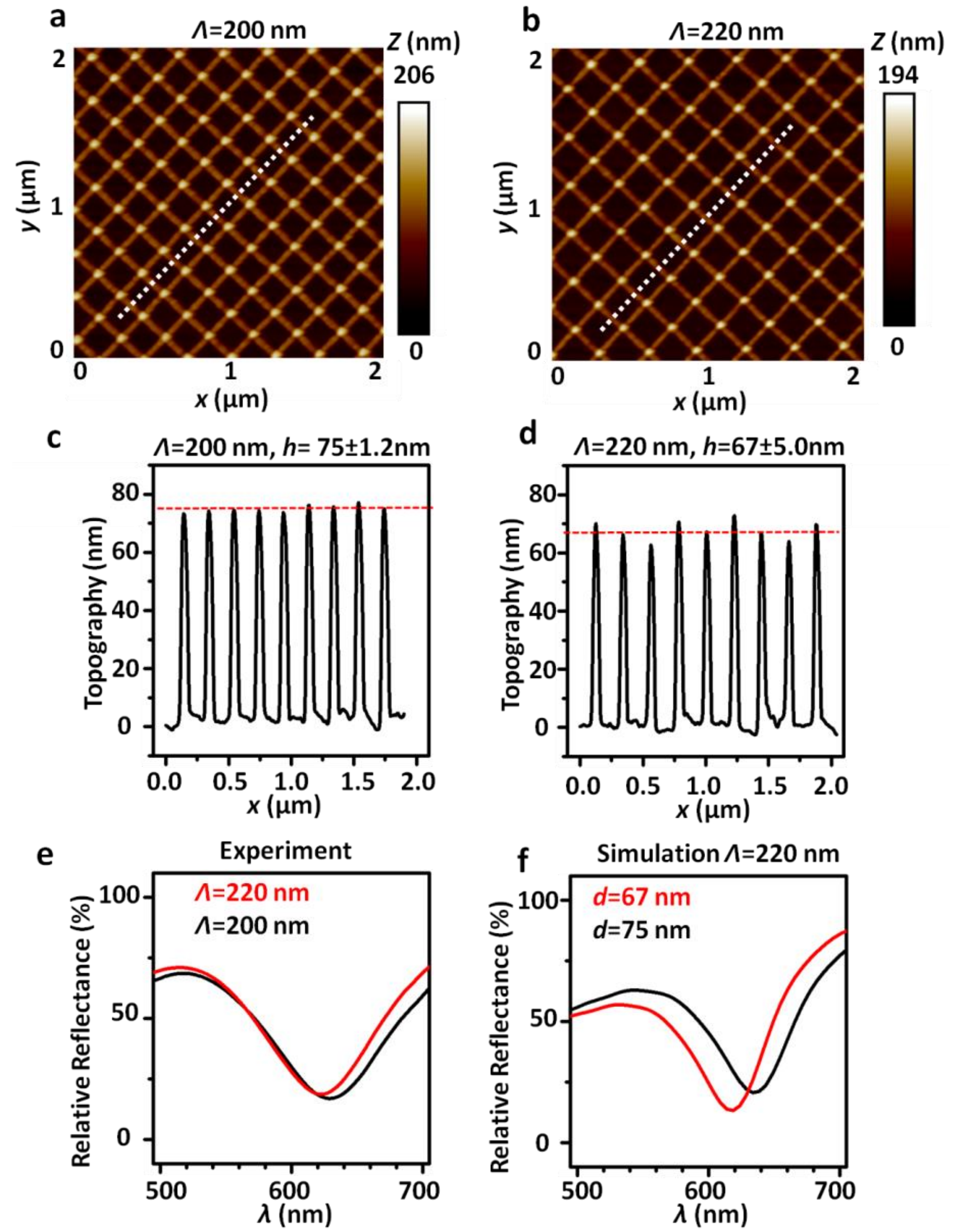

Supplementary Figure 3 | Comparison of nanostructures with pitch size of 200 and 220 nm. AFM measurements of patterned gold nanostructures with pitch sizes of 200 nm (**a**) and 220 nm (**b**), and the associated height profile along the white dashed lines (**c**) and (**d**). (**e**) Experimentally measured relative reflectance spectrum of nanostructures with pitch sizes of 220 nm and 200 nm. (**f**) Simulated relative reflectance spectrum of patterned gold nanostructures with different trench depths. The pitch size is 220 nm.

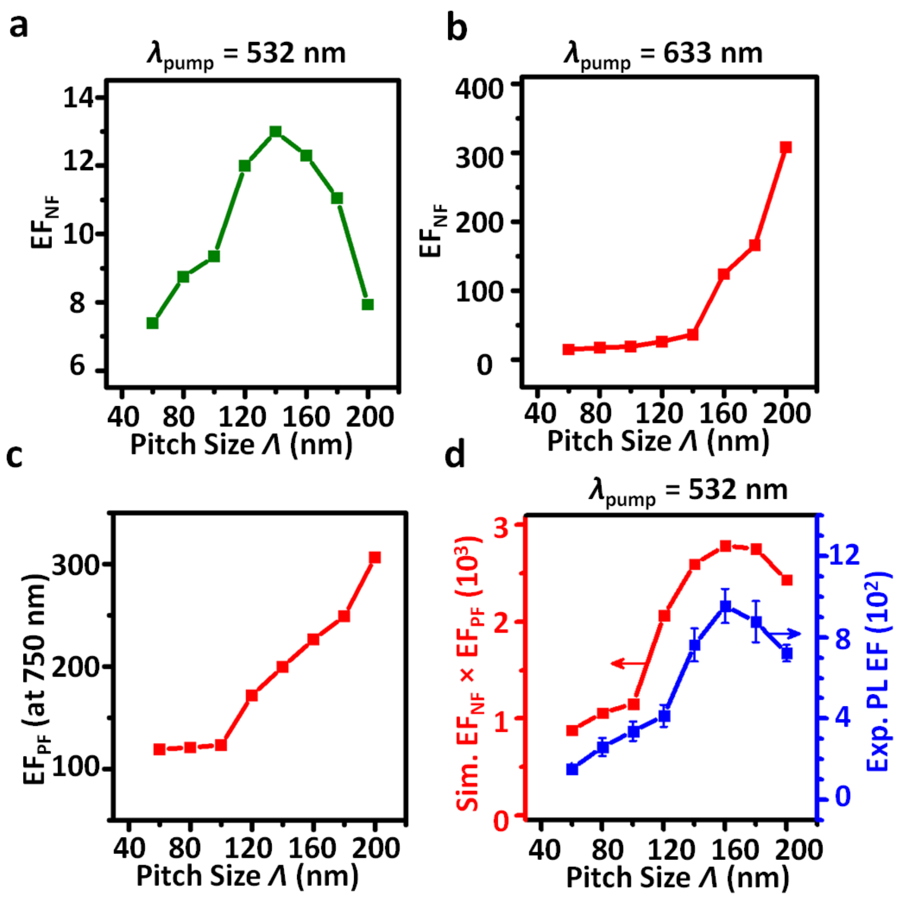

Supplementary Figure 4 | Near-field intensity enhancement, Purcell factor and PL enhancement. Simulated enhancement factor of the near-field intensity for the WSe_2 monolayer flake as placed on the trench region of the gold substrate compared to that on unpatterned gold film, at the pump laser wavelength of 532 nm (**a**) and 633 nm (**b**). This enhancement factor for the near-field intensity is denoted as "EF_{NF}", which accounts for the excitation process. (**c**) Simulated enhancement factor for the Purcell factor at the PL emission wavelength of WSe_2 (750 nm) denoted as "EF_{PF}", accounting for the emission process. (**d**) Comparison of simulated and experimental PL EF excited by 532-nm laser. Here, the experimental PL EF was corrected for the trench area fraction. The simulated PL EF is deduced by the product of EF_{NF} (at 633 nm) and EF_{PF} (at 750 nm). The data points and corresponding error bars (blue) are obtained, respectively, by calculating the mean value and standard error of PL EF based on ten

experimental PL spectra for each nanostructure.

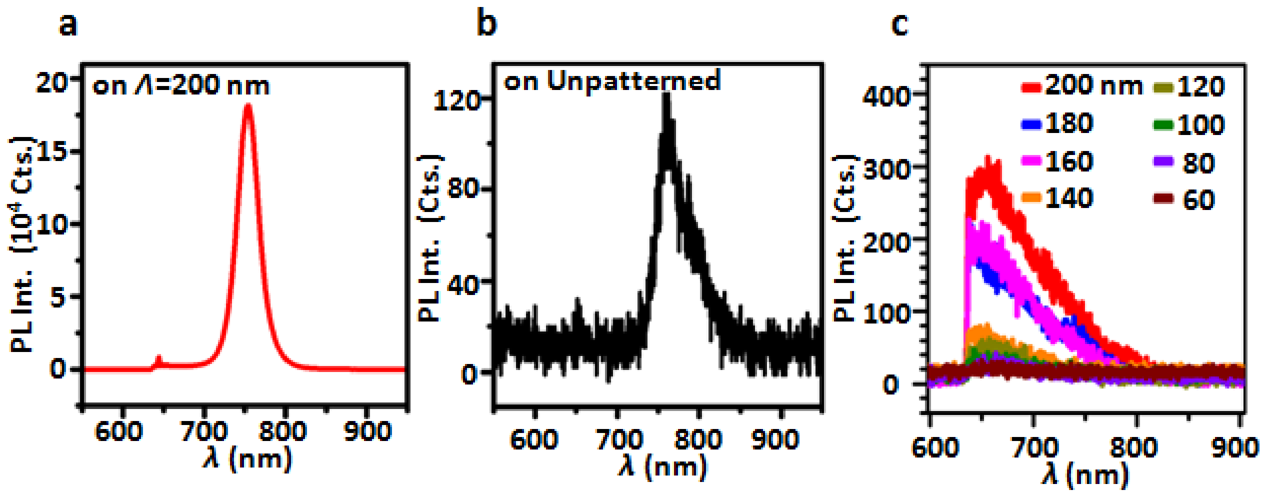

Supplementary Figure 5 | Comparison of PL spectrum of WSe_2 on different substrates and PL spectrum of bare patterned gold nanostructure as measured by 633-nm laser. The long-pass filter in the PL setup cuts off the signal below 633 nm, so there is a sharp slope at 633 nm in each PL spectrum. The PL from WSe_2 on patterned gold nanostructure with pitch of 200 nm in (**a**) was enhanced by up to 1,810-fold (without correction by the trench area fraction) compared to the reference on unpatterned gold film in (**b**)**.** The PL peak energy and full-width at half-maximum (FWHM) in (**a**) are 1.64 eV and 65 meV respectively, while the corresponding values in (**b**) are 1.62 eV and 101 meV. (**c**) PL spectrum as measured from the bare gold nanostructures. Numbers in the figure denote the different pitch sizes in nm. The pump laser power was 30 µW and integration time was 0.5 s.

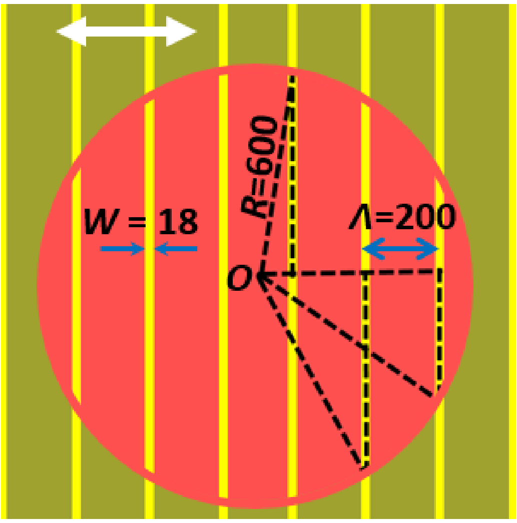

Supplementary Figure 6 | Diagram showing the area of multiple trenches perpendicular to the polarization direction of the laser within a laser spot. The trench parallel to the polarization direction of the laser is not shown for simplicity. The white arrow denotes the polarization direction of the laser and the red circle represents the laser spot. Λ denotes the pitch size and W denotes the trench width. The dashed lines illustrate how Pythagorean theorem was used to calculate the length of the trenches.

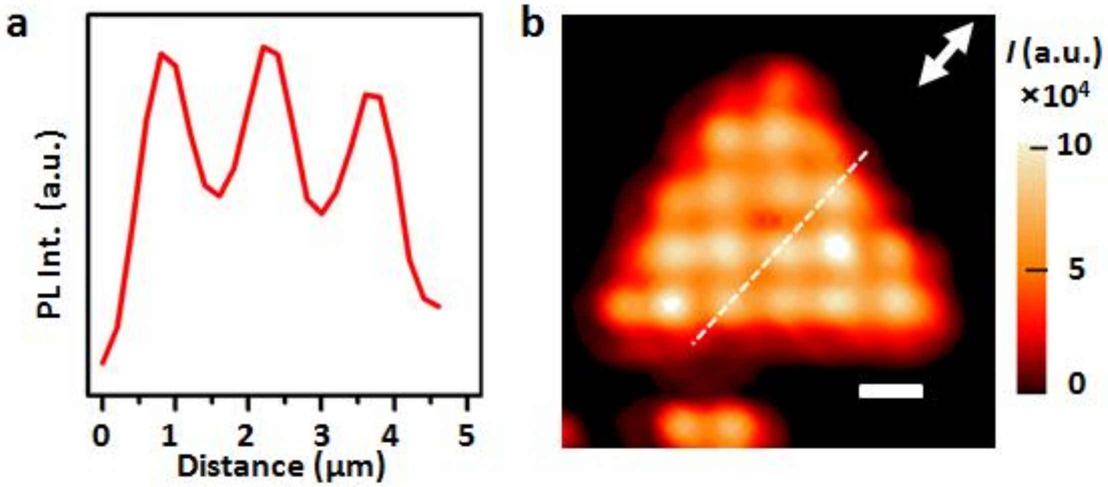

Supplementary Figure 7 | PL intensity as a function of position. PL intensity profile (**a**) along the dashed line as shown in the PL map of (**b**). The excitation laser is 532-nm with an incident polarization of 45 degree as denoted by the white arrow. The scale bar is 1 μm.

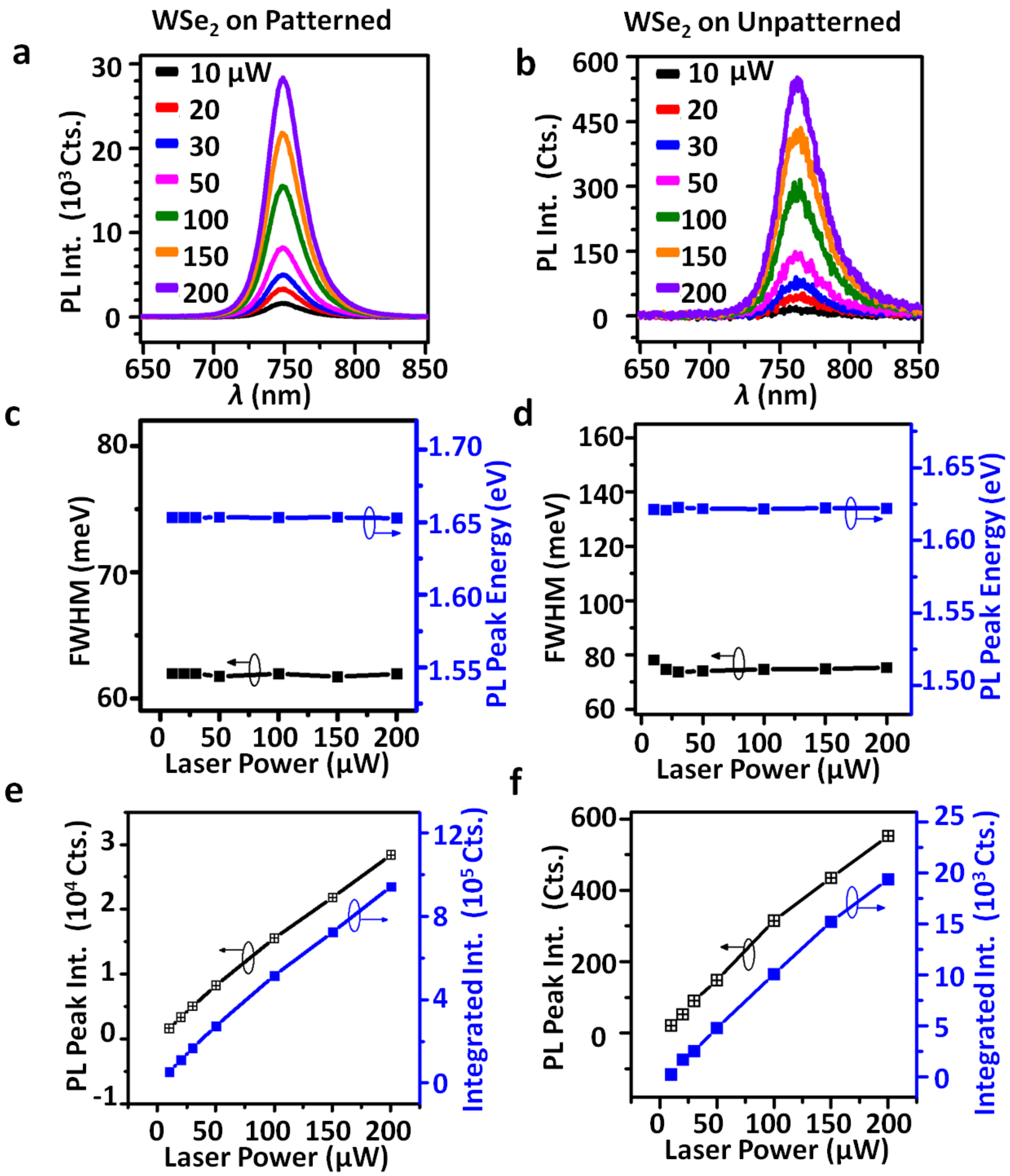

Supplementary Figure 8 | Power dependence of monolayer WSe_2 PL spectra from patterned gold nanostructures (**a**) and unpatterned gold film (**b**), by varying pumping power of 532-nm laser. The integration time in PL measurements was 0.1 s. PL FWHM and peak energy of WSe_2 on the patterned gold substrates (**c**) and on the unpatterned gold film (**d**), as a function of laser power. PL peak intensity and integrated intensity of WSe_2 on the patterned gold nanostructures (**e**) and on the unpatterned gold film (**f**), as a function of laser power.

Supplementary Note

Supplementary Note 1 | Growth and transfer of WSe_2

The WO_3 powders (0.3 g) were placed in a ceramic boat located in the center of the furnace. The Se powders were placed in a separate ceramic boat at the upper stream side maintained at 270 ˚C during the reaction. The sapphire substrates for growing WSe_2 were put at the downstream side, where the Se and WO_3 vapors were brought to the targeting sapphire substrates by an Ar/H_2 flowing gas (Ar = 80 sccm, H_2 = 20 sccm, chamber pressure = 1 Torr). The center heating zone was heated to 925 ˚C at a ramping rate of 25 ˚C /min. Note that the temperature of the sapphire substrates is ~750 to 850 ˚C when the center heating zone reaches 925 ˚C. After reaching 925 ˚C, the heating zone was kept at that temperature for 15 min and the furnace was then naturally cooled down to room temperature.

After growth, the WSe_2 was transferred from the sapphire substrate to the gold substrate by a wet transfer method (Reference 1). First, the WSe_2 on sapphire substrate was coated with a layer of PMMA (950K, A3) by spin-coating (step 1: 500 rpm for 10 s; step 2: 2000 rpm for 70 s), followed by baking at 130 ˚C for 2 min. The PMMA at the edge of the sapphire substrate was stroke off by a knife in order to facilitate the following exfoliation of PMMA-capped WSe_2 from the substrate. Then, a NaOH (3 mol/L) solution at 100 ˚C was used to exfoliate PMMA-capped WSe_2 from sapphire. After that, the PMMA-supported WSe_2 film was transferred to deionized (DI) water so as to remove the etchant and residues. A fresh gold substrate was then used to 'fish out' the PMMA-capped WSe_2 film, followed by drying on a hot-plate (75 ˚C for 5 min and then 100 ˚C for 10 min). The PMMA was finally removed by acetone vapor and cleaned by isopropyl alcohol (IPA).

Supplementary Note 2 | Fabrication process of template-stripped gold substrate

Supplementary Fig. 1 illustrates how the gold substrate consisting of patterned gold structure and unpatterned gold film was prepared by a template-stripping method. First, 2% hydrogen silsesquioxane (HSQ) dissolved in methyl isobutyl ketone (MIBK) was spin coated onto a cleaned silicon substrate at 5000 rounds per minute (rpm). The thickness of the HSQ layer was around 30 nm. Then, the pattern of the HSQ resist was fabricated using electron beam lithography (EBL) with an electron acceleration voltage of 100 keV. The exposed HSQ was developed using NaOH/NaCl (1 wt %/4 wt %) in de-ionized water for 60 seconds before immersing in DI water for 60 seconds to stop the development. Next, silicon etching was carried out by inductively-coupled plasma (ICP) using a gas combination of Cl_2 and HBr. The HSQ feature functioned as a protection mask. After the silicon etching process, the HSQ mask was removed by using reactive-ion-etching (RIE) oxygen plasma, where the HSQ mask was sputtered away by the reactive ions. Next, a 150-nm-thick gold film was evaporated onto the silicon substrate. Several droplets of the optical adhesive (OA) glue were put onto the sample surface using a pipette. A glass slide was then placed on top of the OA glue and the OA glue was cured using ultra-violet radiation, followed by template-stripping process, where the gold substrate was stripped from the silicon template.

Supplementary Note 3 | Comparison of nanostructures with pitch size of 200 and 220 nm

Supplementary Fig. 3 compares the morphology and reflectance spectra of nanostructures with pitch of 200 and 220 nm. The 220-nm-pitch structures were imperfect due to insufficient exposure dose in the electron-beam lithography process. The AFM scan results (Supplementary Figs. 3a-d) show that the 220-nm-pitch silicon fins were ~10 nm shorter and less regular than that at 200-nm-pitch. This is characteristic of electron-beam exposure of lines close to the cutoff dose, which results in jagged tops with reduced heights. Consequently, the gold substrate with the pitch size of 220 nm had shallower and irregular trenches. This shallower trench explains the blue shift of the resonance wavelength (Supplementary Fig. 3e) instead of the expected redshift if trenches had equal depths. As shown in Supplementary Fig. 3f, simulations confirm the blue shifted resonances for structures with shallower trenches.

Supplementary Note 4 | Calculation of the effective PL enhancement

As shown in Supplementary Fig. 6, the area ratio between the hot-spot area and the laser spot one was calculated in order to calculate the PL enhancement factor of monolayer WSe_2 above the trenches. When the incident optical field is polarized along *x*-direction, the hot-spot area will be determined by the trenches perpendicular to the polarization direction of the laser within the laser spot. The laser spot is represented by the red circle with a radius of R (*i.e.* 600 nm) with center point denoted as "O". The pitch size Λ of the structure is 200 nm and the width of the trench W is 18 nm. The hot-spot area A_{gap} can then be calculated by:

$$A_{\text{gap}} = 2 \times W \times [2 \times (\sqrt{R^2 - 100^2} + \sqrt{R^2 - 300^2} + \sqrt{R^2 - 500^2})] \quad (1)$$

A_{gap} is calculated to be 1.0×10^5 nm^2 and the laser spot area A_0 is $\pi\times600^2$ nm^2. Thus, the area ratio of A_{gap}/A_0 calculated by this approach is 9%. The PL EF of WSe_2 over the trench is scaled by multiplying the reciprocal of this ratio.

Supplementary References

1. Chiu, M. H. *et al*. Spectroscopic signatures for interlayer coupling in MoS_2–WSe_2 van der Waals stacking. *ACS Nano* **8**, 9649–9656 (2014).

2. Dong, Z. *et al*. Second-harmonic generation from sub-5-nm gaps by directed self-assembly of nanoparticles onto template-stripped gold substrates. *Nano Lett.* **15**, 5976–5981 (2015).
